# A return to the genetic heritage of durum wheat to cope with drought heightened by climate change

**DOI:** 10.1371/journal.pone.0196873

**Published:** 2018-05-24

**Authors:** Amor Slama, Elhem Mallek-Maalej, Hatem Ben Mohamed, Thouraya Rhim, Leila Radhouane

**Affiliations:** 1 Science Faculty of Bizerte, Carthage University, Bizerte, Tunisia; 2 Arid and Oases Cropping Laboratory, Arid Regions Institute of Medenine, Medenine, Tunisia; 3 Horticulture Laboratory, National Institute of Agronomic Research, Ariana, Tunisia; 4 Plant Physiology Laboratory, National Institute of Agronomic Research, Ariana, Tunisia; University of Delhi, INDIA

## Abstract

The objective of this work was to perform a comparative analysis of the physiological, biochemical and agronomical parameters of recent and heritage durum wheat cultivars (*Triticum durum* Desf.) under water-deficit conditions. Five cultivars were grown under irrigated (control) and rainfall (stressed) conditions. Different agro-physiological and biochemical parameters were studied: electrolyte leakage, relative water content, chlorophyll fluorescence, proline, soluble sugars, specific peroxidase activity, yield and drought stress indices. It was revealed that a water deficit increased proline content, electrolyte leakage, soluble sugars and specific peroxidase activity and decreased relative water content, fluorescence and grain yield. According to these parameters and drought stress indices, our investigation indicated that old cultivars are the best-adapted to local conditions and showed characteristics of drought tolerance, while recent cultivars showed more drought susceptibility. Therefore, local cultivars of each country should be kept by farmers and plant breeders to preserve their genetic heritage.

## Introduction

Cereals have an important place in food security worldwide, particularly in developed countries. Durum wheat is considered a native species in North Africa in general and in Tunisia in particular [1], where durum wheat is grown mainly in sub-humid and semi-arid lands under rainfall conditions. Under these conditions and with climate change now occurring in North African countries [2], durum wheat production is very low and highly variable. Therefore, a challenge for Tunisia in the coming years is to develop more productive cultivars and to increase durum wheat yield in order to satisfy food requirements. However, Tunisian cereal remains highly dependent on climatic conditions characterized by water scarcity and very frequent drought [3]. Yield loss is primarily due to drought [4]. Several studies have projected increases in drought severity, extent and duration in many parts of the world under climate change [5]. Thus, irrigation might be required in these regions to ensure an economically profitable grain yield for farmers.

To survive, plants exposed to water deficit develop morphological, physiological and biochemical changes [6] and evolve various adaptive mechanisms against dehydration [4]. It has been proposed that the critical feature of tolerance to dehydration depends on the ability to limit membrane damage during drought [6]. Dehydration was also found to decrease the relative water content of plant leaves [7]. To reduce the loss of water, stomata will close in proportion to water stress intensity, which causes progressive limitation of CO_2_ availability in chloroplasts, consequently reducing the CO_2_ to O_2_ ratio and the photosynthetic activity [8]. Photosynthesis is a major source of energy and results from the interaction of many factors, such as chlorophyll fluorescence [9]. This parameter is a potential and simple tool to measure plant response to stress [10] and to evaluate the impact of drought stress on the photosynthetic apparatus [11]. Osmotic adjustment was also considered to be an important mechanism of drought tolerance in plants [12]. In fact, cereals under different environmental stresses accumulate organic solutes with low molecular weights such as sugars, betaine and proline to combat dehydration [13]. Drought stress induces proline biosynthesis and significantly increases proline content in comparison to control plants [14]. According to [15], the amount of soluble sugars, proline concentration and activity of free radical scavenging enzymes increased significantly under stress conditions to combat the accumulation of the reactive oxygen species. All these mechanisms implemented by the plant to fight against the lack of water depend on the species, the cultivar and even the ecotype. To distinguish the different reactions of durum wheat varieties, three old and two recent wheat cultivars were investigated based on certain agro-physiological and biochemical parameters.

## Materials and methods

The plant material used in this work was represented by five durum wheat cultivars: Karim (Ka), Om Rabiaa (Ou), Nasr99 (Na), Maali (Ma) and Salim (Sa). Karim has been cultivated in Tunisia since 1973 and was registered in the official Tunisian catalog in 1982; Om Rabiaa has been cultivated since 1987 and was registered in the catalog in 1996; Nasr 99 has been cultivated since 1990 and was registered in the catalog in 2004; Maali has been cultivated since 1992 and was registered in the catalog in 2007; and Salim is the most recent cultivar, selected from a cross made in Tunisia in 1993, and was registered in the catalog in 2009 [16]. Consequently, these five varieties offer a highly representative basis to compare old and recent durum wheat genotypes.

### Experiment and growth conditions

Experiments were conducted in two growing seasons, 2015–2016 and 2016–2017, under two water regimes: irrigated (T1), referred to as control plants, and rainfall (T2), referred to as stressed plants. The experiment was carried out in the research area of the Field Crops Station of INRAT, which is characterized by semi-arid conditions, and was conducted in two blocks as a completely randomized design for irrigated and rainfall trials. Each block was planted in 15 plots (5 cultivars x 3 replicates). Each plot was planted in four rows of four meters (4 m), spaced 0.25 m apart, with an area of 4 m^2^/plot. Grain density was adjusted to 400 grains/m^2^. In the rainfall trials from December 2015 to May 2016, no irrigation was applied throughout (rainfall = 215 mm). In the irrigated trial, irrigation was applied according to plant necessity during the crop life-cycle (approximately 400 mm). Four irrigation treatments were applied, 45 mm each, in December, February, April and May 2015–2016 (rainfall + irrigation: 215 mm + 180 mm = 395 mm). In the second season, from December 2016 to May 2017, the rainfall = 187 mm, and four irrigation applications of 50 mm each were applied to the irrigated trials in February, March, April and May (rainfall + irrigation: 187 mm + 200 mm = 387 mm).

Four parameters were studied in the first season (2015–2016): electrolyte leakage, relative water content, chlorophyll fluorescence and proline content. To confirm results obtained in the first season, in the second growing season (2016–2017), other criteria were studied: soluble sugar content, specific peroxidase activity and grain yield which is used to calculate several drought stress indices.

### Electrolyte leakage

Membrane integrity was estimated at heading stage during the 2015–2016 season by measurement of electrolyte leakage using a conductometer. Ten leaf segments of 1 cm^2^ were cut from uppermost fully expanded leaves obtained from the two water treatments (T1: control treatment, and T2: stressed treatment). Segments were rinsed 3 times in order to eliminate surface electrolytes and placed in tubes containing 5 mL of demineralized water in the refrigerator. After 24 hours, the free conductivity of the solution (FC1: control, FC2: stressed) was determined at 25°C. Tubes were then placed in a water bath for 1 h at 90°C to destroy the cell membranes. The total conductivity of the solution (TC1: control, TC2: stressed) was then determined at 25°C. The electrolyte leakage was calculated as $\frac{FC}{TC}\times 100$ [17].

### Relative water content

Relative water content (RWC) of stressed plants leaves was measured at heading stage during the 2015–2016 season. One leaf from the uppermost fully expanded leaves of each cultivar was detached and covered to avoid water loss, then weighed immediately (fresh weight = FW). Leaves were kept for 48 h in Petri dishes on filter-paper discs moistened with distilled water in the refrigerator to determine their turgid weight (TW). Leaves were then kept at room temperature (25° C) for free transpiration. The weight of these excised leaves was recorded, respectively, every one minute for a period of 10 min, every five minutes for a period of 50 min and every fifteen minutes for a period of 30 min. The dry weight (DW) was measured after drying leaves for 48 h at 70°C. RWC was determined as follows:

$$RWC\;(\%)=\frac{FW\;-DW}{TW-DW}\times 100$$

### Chlorophyll fluorescence

The chlorophyll fluorescence of the youngest fully expanded leaf was measured at heading stage during the 2015–2016 season using a portable fluorometer (Hansatech Instruments Handy, PEA). The leaf was dark adapted for 15 min to determine the initial fluorescence (F0), then a saturating flash of light was used to measure the maximal fluorescence (Fm). Chlorophyll fluorescence was determined by the ratio $\frac{Fv}{Fm}$.

### Proline content

Proline content was determined at heading stage during the 2015–2016 season using the ninhydrin method according to [18]. For each replication, 200 mg of fresh leaves was used and was divided into two parts: 100 mg was used to determine the dry matter weight after oven drying at 70°C for 48 h; the second part was used to measure proline content. The spectrophotometric absorbance was read at 528 nm and proline content calculated to μg/g dry matter.

### Specific peroxidase activity

Specific peroxidase activity (SPA) in the flag leaf was measured at the heading stage during the second season, 2016–2017. A total of 200 mg of the fresh leaves per replicate was divided into two parts: 100 mg was used to determine the dry matter weight; the second part was used to measure the SPA. Following [19], 100 mg was frozen in liquid nitrogen, and the powder was suspended in solution composed of 50 mM phosphate buffer at pH 7, 100 mM KCl, 1 M NaCl, 1 mM CaCl_2_, 0.1% Triton X-100 and 1% PVP. The homogenate was centrifuged at 15000 g for 30 min at 4°C, after which the samples of each cultivar were stored at -20°C until use. To determine the specific peroxidase activity, 1 mL of this solution in the presence of 0.1 M of phosphate (pH 7), 0.1% of guaiacol and 30% of H_2_O_2_ were used to follow the change of absorbance at 470 nm for 12 minutes. The results were expressed by μM mg ^-1^ mn-^1^.

### Soluble sugars

Total soluble sugar content was determined at heading stage in the 2016–2017 season by anthrone sulfuric acid extraction according to the method in [20]. An anthrone reagent was prepared with 0.2 g anthrone, 8 mL absolute ethyl alcohol, 30 mL distilled water and 100 mL sulfuric acid. A total of 100 mg dry weight powder of the flag leaf was boiled for 1 h, cooled, and filtrated, after which 0.5 mL of the extract was mixed with 4.5 mL of the anthrone reagent. The absorbance was measured at 625 nm using glucose as a standard, and the results were expressed by μM g^−1^ dry matter.

### Yield and drought stress indices

Grain yield was measured at the second season 2016–2017. The Drought stress indices: Mean Productivity (MP), Stress Susceptibility Index (SSI) and Stress Tolerance Index (STI) were measured according to [21].

- $MP=\frac{GYr+GYi}{2}$
  GYr: grain yield under rainfall conditions
  GYi: grain yield under irrigated conditions.
- $STI=\frac{GYi\times GYr}{({MGYi)}^{2}}$
  MGYi: mean grain yield of all cultivars under irrigated conditions.
- $SSI=\frac{1-\;\frac{\;GYr}{GYi}}{SI},\;SI=1-\frac{MGYr}{MGYi}$
  MGYr: mean grain yield of all cultivars under rainfall conditions.

### Data analysis

The statistical analysis was performed with SAS, statistical software version 6.12 (SAS Institute, Cary, NC, U.S.A). Mean values were obtained as a mean of triplicate analysis. ANOVA (Analysis of variance) was used to compare the means. Differences were considered significant at p < 0.05 using Duncan’s Multiple Range test.

## Results and discussion

Under water deficit conditions, plants present a series of morpho-physiological and biochemical changes as part of strategies to reduce the water stress effects.

### Effect of water deficit on membrane integrity

Measurement of electrolyte leakage was considered a typical method to estimate membrane integrity in response to environmental stresses and appeared to be a relevant criterion [22]. Table 1 showed that membrane integrity was affected by water stress. The rise of electrolyte leakage observed in this condition was attributed to disruption of cell membranes, probably resulting from protein degradation [22]. Furthermore, the present study showed significant variation among genotypes. Karim and Nasr99, the oldest cultivars, which exhibit the lowest values of electrolyte leakage, were the two most resistant cultivars, while Salim, the most recent, was one of the most susceptible cultivars.

**Table 1:** Electrolyte leakage (%) and proline content (μg/g DM) at heading stage according to cultivars and treatments during the 2015–2016 season.

Cultivars		Ka	Na	Ou	Ma	Sa
**Electrolyte leakage**	T1	11.19^b^	15.13^a^	10.25^b^	9.81^b^	9.35^b^
	T2	32.57^b^	30.91^b^	33.56^b^	39.02^a^	37.37^a^
	Increase (%)	x 2.91	x 2.04	x 3.27	x 3.97	x 3.99
**proline content**	T1	443^d^	1032^b^	809^c^	2786^a^	2546^a^
	T2	2567^d^	4986^c^	2792^d^	7849^a^	5054^b^
	Increase (%)	x 6	x 5	x 3.5	x 3	x 2

Karim (Ka), Om Rabiaa (Ou), Nasr99 (Na), Maali (Ma) and Salim (Sa). T1: irrigated, T2: rainfall. DM: dry matter.

Different letters (a, b, c, d) indicate significant differences among cultivars according to Duncan’s multiple range test (P < 0.05).

### Effect of water deficit on relative water content

**Fig 1:**
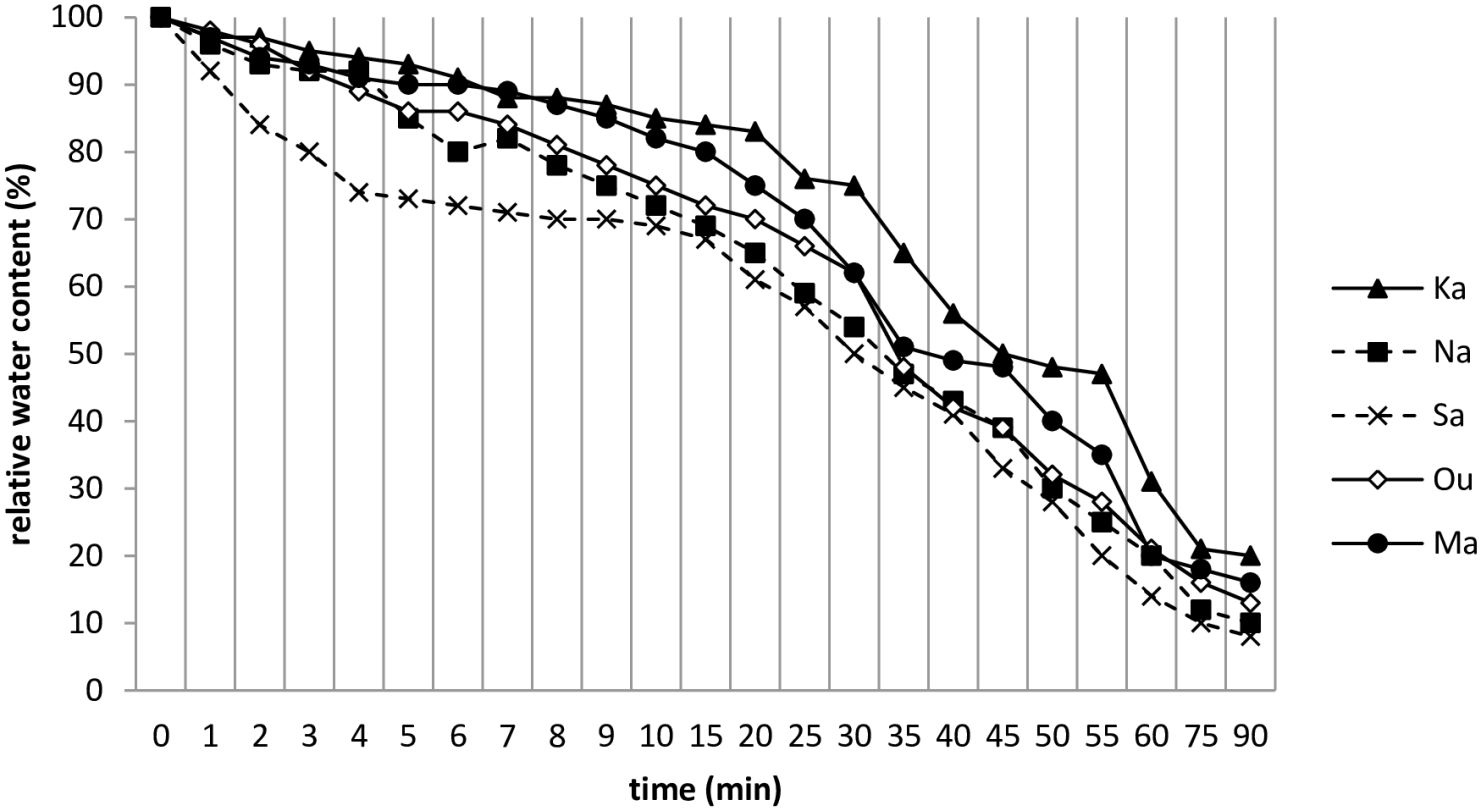
Variation of relative water content by time (min) of durum wheat cultivars during the 2015–2016 season. Karim (Ka), Om Rabiaa (Ou), Nasr99 (Na), Maali (Ma) and Salim (Sa).

Relative water content (RWC) measurement was a general method used to determine leaf-water balance in plants during water-deficit periods [23]. Water loss in the stressed leaves of durum wheat was expressed as the leaf water loss rate calculated as a percentage of RWC (%) decrease by time (min) (Fig 1). The general trend of curves throughout showed that Karim had low water loss compared to Salim, which showed an important decease in RWC. Fig 1 also shows a decrease in the RWC in leaves from 100% at the initiation of experience to 20%, 16%, 13%, 10% and 8% at 90 min for Karim, Nasr99, Om Rabiaa, Maali and Salim, respectively. The highest final RWC was revealed in Karim, and the lowest value was observed in the Salim cultivar. This parameter allowed a classification similar to those obtained by electrolyte leakage: Karim was one of the most resistant, while Salim was the most sensitive to drought.

### Effect of water deficit on chlorophyll fluorescence

**Fig 2:**
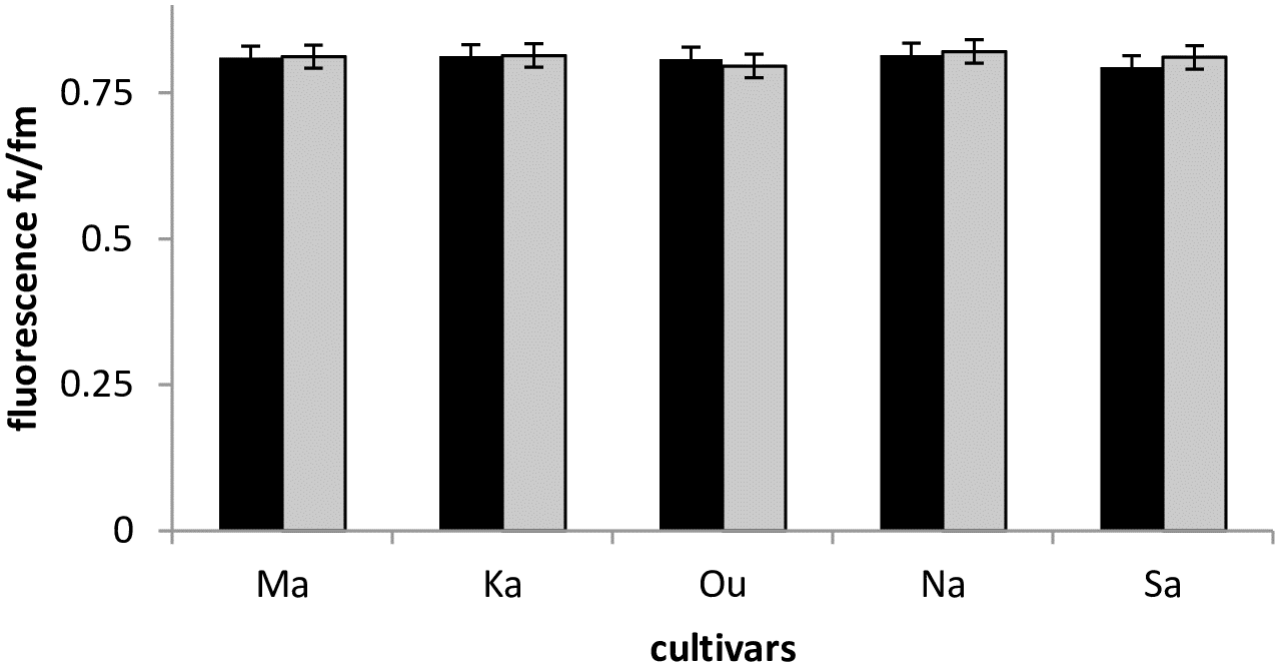
Variation of Fv /Fm according to durum wheat cultivars and treatments at the heading stage during the 2015–2016 season. Rainfall: black bar, irrigated: grey bar, Karim (Ka), Om Rabiaa (Ou), Nasr99 (Na), Maali (Ma) and Salim (Sa).

Because it is a fast, powerful, simple and non-destructive technique to measure plant response to abiotic stress, chlorophyll fluorescence is often used in applied research and practice [10, 24]. The Fv/Fm ratio represents the photochemical capacity of photosystem II (PSII) [25]. Therefore, a high value of this ratio indicates that the photosystem is in good condition, whereas lower values of this ratio signify a PSII-center injury under drought conditions. In response to water stress, our study showed a reduced value of Fv/Fm, under 0.830 (Fig 2). The substantial Fv/Fm decrease was a result of the decreased ability of PSII to reduce the primary acceptor QA (Quinone A) [26]. Our finding was in agreement with previous results that showed that Fv/Fm of drought-stressed plants was lower than that of plants growing in optimal environmental conditions [4, 8, 26, 27]. Compared to the control treatment, Fv/Fm was significantly lower for Karim, Nasr99, Maali and Salim by 0.2%, 0.3%, 0.8% and 2.4%, respectively. Om Rabiaa, on the other hand, exhibited an Fv/Fm ratio increase of 1.5%, making it the most resistant cultivar, followed by Karim and Nasr99.

### Effect of water deficit on proline content

The synthesis of proline has been reported to occur in response to many stresses, including drought [28]. Compared to control plants, the foliar proline in our studied cultivars (Table 1) showed an increase in all stressed plants. Rainfall conditions induced an increase in proline content similar to changes observed in electrolyte leakage, but with higher (6-fold) amplitude. Similar results were reported by [29], which reported that proline content was positively correlated with membrane integrity, suggesting its use as a selection criterion for drought tolerance. This seems to indicate that proline may play a protective role in minimizing the damage caused by dehydration [30].

Proline content was found to respond differently to drought depending on the studied cultivar (Table 1). The proline content was approximately 2-fold in recent cultivars but was much more pronounced in the old cultivars, peaking at approximately 6-fold for Karim, the most significant increase; this allowed Karim a good ability to overcome the impact of water stress. In contrast, Salim had the lowest proline increase (x2), making it the most sensitive cultivar. These results were in agreement with [31] and [14], which reported that drought stress induced proline biosynthesis and significantly increased proline content in leaves by 86.9% to 700% in comparison with control plants.

Our findings established two groups of cultivars with respect to proline accumulation (Table 1), each with a different resistance mechanism for water deficit. The old cultivars (Om Rabiaa, Nasr99 and Karim) appeared to be more reactive, with a high rate of proline accumulation at water stress conditions (3.5-, 5- and 6-fold more elevated), while recent cultivars (Maali and Salim) were characterized by a particular strategy of prevention, with a higher proline content even at well-watered conditions. These results were in accordance with previous indications that the older cultivars accumulated higher levels of proline than the recent cultivars under water stress [13]. Our older cultivars had the capacity to osmotically adjust and to resist drought, whereas for the recent cultivars, the role of proline during stress played a role in preventing hyperosmotic stress [30].

### Effect of water deficit on soluble sugars

Leaf osmotic adjustment via soluble sugars has been known to be a mechanism to resist water-deficit stress in many plants [32]. The study of [33] indicated that an increase in drought stress intensity from well-watered treatment to very severe drought leads to increased leaf-soluble sugar content. In [15], a 33% increase in soluble sugar content was observed in stressed plants. Our investigation showed that soluble sugars were positively affected by water deficit, with an increase of 18.39% (Table 2). The oldest cultivars–Om Rabiaa, Karim and Nasr99 –showed the highest increases (26.15%, 23.62% and 21.67%, respectively), followed by the modern cultivars Salim and Maali (11.77% and 8.78%, respectively).

**Table 2:** Soluble sugar content (μM g^−1^ DM), specific peroxidase activity (μM mg^-1^ mn^-1^), grain yield (Q/ha) and drought stress indices during the 2016–2017 season.

Cultivars		Ka	Na	Ou	Ma	Sa
**Soluble sugars**	T1	84.33^a^	75.26^b^	78.35^ab^	79.23^ab^	77.52^ab^
	T2	104.25^a^	91.57^bc^	98.84^ab^	88.56^c^	84.33^c^
	Increase (%)	23.62	21.67	26.15	11.77	8.78
**Specific peroxidase activity**	T1	0.675^d^	0.742^cd^	1.202^a^	1.033^b^	0.821^c^
	T2	1.040^bc^	0.913^c^	1.501^a^	1.205^b^	0.991^c^
	Increase (%)	54.07	23.04	24.87	14.27	20.7
**Grain yield**	T1	38.92^a^	29.29^c^	31.56^b^	29.28^c^	27.76^c^
	T2	32.45^a^	26.32^b^	25.33^b^	19.85^c^	20.39^c^
	Decrease (%)	16.62	10.13	19.74	32.20	26.54
**Stress indices**	MP	35.68^a^	27.80^b^	28.44^b^	24.56^c^	24.07^c^
	STI	1.28^a^	0.78^bc^	0.81^b^	0.59^bc^	0.57^c^
	SSI	0.80^bc^	0.49^c^	0.95^bc^	1.60^a^	1.28^ab^

Karim (Ka), Om Rabiaa (Ou), Nasr99 (Na), Maali (Ma) and Salim (Sa), irrigated conditions (T1), rainfall conditions (T2), Mean Productivity (MP), Stress Susceptibility Index (SSI) and Stress Tolerance Index (STI). DM: dry matter.

Different letters (a, b, c, d) indicate significant differences among cultivars according to Duncan’s multiple range test (P < 0.05).

### Effect of water deficit on specific peroxidase activity

Reactive oxygen species generated in stressed plant cells were highly cytotoxic. These compounds were controlled by different antioxidant defense system enzymes. Different abiotic stressors led to increased antioxidant enzymes. [34] showed that peroxidase activity increased significantly with the increase in lead concentrations, from 50% to more than 100% at high lead concentrations. The results from [15] indicated high peroxidase activity under moderate drought conditions. Our study supports these results by showing an average 27.39% enhancement of peroxidase activity under water-deficiency conditions (Table 2).

Regardless of the water regime, the Om Rabiaa (old) and Maali (recent) cultivars had the highest specific peroxidase activity. Regarding the increase in specific peroxidase activity in a limited water regime (Table 2), Karim cultivar was the most resistant to water deficit conditions (54.07%), while the most affected cultivar was Maali, with a small increase in specific peroxidase activity (14.27%). This parameter allowed a classification similar to those obtained by proline content. The old cultivars appeared to be more reactive, with a high rate of peroxidase activity at water stress conditions, while recent cultivars were characterized by a prevention strategy.

### Grain yield and drought stress indices

A high grain yield is a major goal for the improvement of durum wheat, particularly in drought areas [35]. Our results showed that grain yield was significantly higher under irrigated conditions (Table 2). Excepting the Karim cultivar, which had the highest grain yield, no difference was seen among the other cultivars in well-watered conditions. Statistical data also showed that not all old cultivars had high yields in irrigated conditions. Duncan’s Multiple Range Test (Table 2) divided all cultivars in 3 groups. The Karim cultivar also had the most elevated grain yield under low water availability. On average, water stress decreased yield by 21.04%. The lowest decrease in grain yield was observed for the old cultivars Nasr99, Karim and Om Rabiaa (10.13%, 16.62% and 19.74%, respectively), making them the most resistant to drought; the modern cultivars Salim and Maali, on the other hand, can be perceived as more sensitive to drought, as they showed decreases of 26.54% and 32.20%, respectively.

According to [1], a history of wheat breeding in Tunisia, Karim had an excellent capacity to adapt and a high yield under a wide range of conditions, reaching more than 60% of the national durum wheat area within a few years of its release in 1973. The next released cultivar was Om Rabiaa, which was selected for its good drought tolerance and good straw yield for the semi-arid region. Nasr99 has the highest yield potential under favorable conditions and under water-limited conditions. The area cultivated with Maali is rapidly increasing a few years after the selection, especially in the more humid areas, but not much progress has been made under rainfall conditions. Seed production of Salim started in 2010 and few data were available for this cultivar. Our results showed that genotypes with high grain yields were associated with low effects from water deficit. These results are in agreement with those of [36], which reported a close relationship between high water-retention capability, drought hardiness and high yield in wheat. Moreover, the higher values of grain yield recorded in our study for the oldest cultivars confirm the high accumulation of proline, sugars and peroxidase activity, low electrolyte leakage and slight decrease in Fv/Fm and RWC. Consequently, cultivars able to tolerate water deficit must therefore be able to increase their grain yield. Understanding the physiological basis of yield formation is a fundamental step to providing a more holistic view for plant breeders and increasing the potential productivity of cereals [37].

According to stress indices (MP, STI and SSI) the group of older cultivars was determined to also be tolerant, whereas recent genotypes were found to be sensitive to water deficit (Table 2). The high values of MP and STI were indicative of plant tolerance to water deficiency. The MP and STI were more elevated for the first group, while the SSI recorded for Salim and Maali showed high values.

## Conclusions

The objective of this work was to investigate the morpho-physiological and biochemical responses of durum wheat cultivars to water stress and to evaluate the contrasting physiological responses between old and recent cultivars. When the effects of water deficit were examined, old cultivars were found to have the highest grain yield, whereas recent cultivars performed weakly under these conditions. Old cultivars have been found to be highly productive cultivars and had traits of resistance to drought since their creation; these characteristics were improved over time because these cultivars have originally and over the long-term adapted to their environment. However, the recently created cultivars Maali and Salim have not responded positively to local climatic conditions, despite the many years of testing by breeders and farmers, and this is probably due to an absence of drought traits in them.

Therefore, created new genotypes or introduced exotic ones, should not prevent countries from maintaining their own local cultivars. The culture of old cultivars should be prioritized as a means to preserve genetic heritage. Local cultivars represent a very important reserve of genes that can be introduced into other varieties for improving adaptation to abiotic stresses such as drought.
